# Diet affects glycosylation of serum proteins in women at risk for cardiometabolic disease

**DOI:** 10.1007/s00394-021-02539-7

**Published:** 2021-03-26

**Authors:** Tyler Kim, Yixuan Xie, Qiongyu Li, Virginia M. Artegoitia, Carlito B. Lebrilla, Nancy L. Keim, Sean H. Adams, Sridevi Krishnan

**Affiliations:** 1 Global Disease Biology, University of California Davis, Davis, USA; 2 Department of Chemistry, University of California Davis, Davis, USA; 3 Obesity and Metabolism Research Unit, USDA-WHNRC, 430 W. Health Sciences Drive, Davis, CA 95616 USA; 4 Department of Nutrition, University of California Davis, Davis, USA; 5 Department of Surgery, University of California Davis School of Medicine, Sacramento, USA; 6 Center for Alimentary and Metabolic Science, University of California Davis School of Medicine, Sacramento, USA

**Keywords:** Post-translational modification, Glycosylation, Dietary Guidelines for Americans, Sialylation, Fucosylation, Glycan, Glycoproteomics

## Abstract

**Background:**

Glycoproteomics deals with glycoproteins that are formed by post-translational modification when sugars (like fucose and sialic acid) are attached to protein. Glycosylation of proteins influences function, but whether glycosylation is altered by diet is unknown.

**Objective:**

To evaluate the effect of consuming a diet based on the Dietary Guidelines for Americans on circulating glycoproteins that have previously been associated with cardiometabolic diseases.

**Design:**

Forty-four women, with one or more metabolic syndrome characteristics, completed an 8-week randomized controlled feeding intervention (*n* = 22) consuming a diet based on the Dietary Guidelines for Americans (DGA 2010); the remaining consumed a ‘typical American diet’ (TAD, *n* = 22). Fasting serum samples were obtained at week0 (baseline) and week8 (post-intervention); 17 serum proteins were chosen for targeted analyses. Protein standards and serum samples were analyzed in a UHPLC-MS protocol to determine peptide concentration and their glycan (fucosylation or sialylation) profiles. Data at baseline were used in correlational analyses; change in proteins and glycans following intervention were used in non-parametric analyses.

**Results:**

At baseline, women with more metabolic syndrome characteristics had more fucosylation (total di-fucosylated proteins: *p* = 0.045) compared to women with a lesser number of metabolic syndrome characteristics. Dietary refined grain intake was associated with increased total fucosylation (*ρ* = − 0.530, *p* < 0.001) and reduced total sialylation (*ρ* = 0.311, *p* = 0.042). After the 8-week intervention, there was higher sialylation following the DGA diet (Total di-sialylated protein *p* = 0.018, poly-sialylated orosomucoid *p* = 0.012) compared to the TAD diet.

**Conclusions:**

Based on this study, glycosylation of proteins is likely affected by dietary patterns; higher sialylation was associated with a healthier diet pattern. Altered glycosylation is associated with several diseases, particularly cancer and type 2 diabetes, and this study raises the possibility that diet may influence disease state by altering glycosylation.

**Clinical trial registration:**

NCT02298725 at clinicaltrials.gov; https://clinicaltrials.gov/ct2/show/NCT02298725.

**Supplementary Information:**

The online version contains supplementary material available at 10.1007/s00394-021-02539-7.

## Introduction

The Dietary Guidelines for Americans (DGA), a resource that guides what constitutes a healthy diet, is aimed at preventing chronic illnesses [1]. These guidelines have only been tested twice in a controlled feeding setting, which is considered the gold standard in nutrition research [2]. Schroeder et.al. evaluated the effect of consuming a DGA-based diet for 4-weeks on circulating lipids and glucose [3]. We recently evaluated the effect of consuming a DGA based diet for twice that duration (8 weeks) on insulin resistance and circulating lipids [4]. Neither study reported changes in their primary lipid or glucose metabolism outcomes related to cardiometabolic health. While recent efforts have focused on identifying metabolomics-based biomarkers of healthy dietary patterns in large-scale epidemiological studies [5], no study to date has reported changes in metabolomics biomarkers following a controlled feeding trial of the Dietary Guidelines for Americans. Metabolomics, the study of small molecules, has become an important aspect of understanding the impact of diet on health, not to mention a key approach to achieving precision nutrition [6]. Both lipidomics and proteomics have recently become popular in nutrition research [7]. In the current report, we used targeted glycoproteomic analyses, a branch of proteomics that measures glycoproteins (proteins with sugars or glycans bonded to specific amino acid residues), to evaluate selected serum proteins that are associated with cardiometabolic disease risks.

The glycoproteome, which encompasses the profile of glycans and their protein sites, is a result of post-translational modification (PTM) of proteins. PTM is an important cellular process that begins in the Golgi body and ends in the endoplasmic reticulum (ER) [8, 9], influencing various aspects of protein folding and function as well as protein trafficking pathways [10]. Glycoproteins are crucial to a host of cellular mechanisms, all of which are related to the maintenance of health including cell signaling, protein recognition, and promotion or inhibition of endocytosis [11]. The perturbation of any of these processes can lead to the development of metabolic diseases [12, 13]. The glycoproteome has been recognized in the past decade to be involved in several diseases, including cancer, type 2 diabetes [14], neurodegenerative disease, and chronic metabolic diseases [15]. Sialylated (the addition of 9-carbon sugar: e.g., neuraminic acid, as a terminal glycan) and fucosylated (addition of 5-carbon fucose as a terminal glycan) proteins are being investigated as primary players in discriminating between diseased and healthy states. Fucosylated proteins have recently begun to be used as biomarkers for specific types of cancers [16], and the inter-individual variability in sialylated glucose transporter proteins has recently been associated with plasma glucose control [14]. Sialylated proteins play crucial roles in innate immunity ranging from antigen detection to maintaining the balance between suppression or activation of immune responses [17]. In two previous observational studies, we evaluated associations between glycosylation of HDL-associated proteins and coronary artery disease, chronic renal disease, or metabolic syndrome [18, 19]. We reported that greater sialylation of proteins was associated with reduced severity of metabolic disease [19].

Even though the glycoproteome is being studied in cancer biology and immunology, the effect of diet on these glycans has not been evaluated thus far. To address this knowledge gap, we chose to leverage a controlled feeding clinical trial completed at the Western Human Nutrition Research Center in 2018 (NCT02298725 at clinicaltrials.gov). In this report, we present glycoproteome data at baseline (before the intervention began) in women who had one or more characteristics of metabolic syndrome, as well as following an 8-week controlled feeding intervention where the women either followed a diet based on the Dietary Guidelines for Americans 2010 (DGA) or a typical American diet (TAD). Women with metabolic syndrome were chosen because this condition is coincident with increased risk for type 2 diabetes and cardiovascular mortality [20]. Further, the etiology and pathogenic manifestations of metabolic syndrome are different between the sexes. Based on NHANES data between 1988 and 2006, age-adjusted rates for the prevalence of metabolic syndrome in the U.S. increased more in women (by 6%) compared to men, suggesting women are at higher risk [21]. An 8-week intervention was chosen as an optimal duration to evaluate the effect of the dietary pattern, in the absence of energy deficit or weight loss, even though lipid changes might show up after 3–4 weeks [22], hemoglobin A1c would require at least 8 weeks to show significant changes [23]. The two aims that will be addressed in this report are the following: (a) to establish associations between baseline habitual diet, clinical characteristics, and glycome profile of select serum proteins that have been associated with chronic metabolic diseases; and (b) to identify the effect of an 8-week exposure of a DGA or TAD diet on the serum glycoproteome profile of women at-risk for metabolic disease.

## Methods

### Clinical trial overview

The clinical trial from which this report originates is registered as NCT02298725 at clinicaltrials.gov. Details regarding recruitment, place of study, randomization, inclusion, and exclusion criteria for participants, blinding, and description of primary and secondary endpoints have been published [4]. The study was conducted at the USDA/ARS-Western Human Nutrition Research Center (WHNRC) on the campus of the University of California Davis (UC Davis). The study was approved by the UC Davis Institutional Review Board. Participants provided written informed consent for participating in the study (IRB approval number: 648620-12).

### Study participants

Forty-four women (age 20–65 years) with BMI between 25.1 and 39.9 kg/m^2^, resting blood pressure ≤ 140/90 mm Hg, and one or more characteristics of metabolic syndrome (fasting glucose ≥ 100 and < 126 mg/dL; oral-glucose-tolerance test (OGTT) 2-h glucose > 140 and < 199 mg/dL; Quantitative Insulin Sensitivity Check Index (QUICKI) score < 0.315, homeostasis model assessment (HOMA) > 3.67, or log HOMA > 0.085; or HbA1c ≥ 5.7 and < 6.5) were recruited and enrolled in this double-blind randomized (block randomization, blocks of 2, with a 1:1 allocation ratio) 8-week controlled feeding clinical trial and completed the intervention. Of the 44, 22 women were in a group that was given a diet based on the Dietary Guidelines for Americans 2010 (DGA group), and the remaining 22 consumed a diet that resembled a typical American diet (TAD group) based on National Health and Nutrition Examination Survey data. Women were tested before and after the feeding trial, and fasting serum samples were used in the current report.

### A brief overview of the dietary intervention

In-depth information about the diet intervention including individual day menus are published in a methods manuscript outlining the process of designing, blinding the intervention from participants, and delivering the diets in this randomized controlled intervention [24]. Briefly, the energy balanced diets were designed to fit into one that matches the DGA pattern (2.3 cups fruits, 3.5cups of vegetables, 2.8 oz of whole grains, 2.4 oz of refined grains, 1.1 oz of seafood, 3.4 oz of meat, poultry and eggs, 0.8 oz of nuts and seeds, 3.3 cups of dairy and 15% total energy intake from solid fats and added sugars), and another that matches a TAD pattern (1 cup of fruits, 1.5 cups of vegetables, 1.1 oz of whole grains, 5.7 oz of refined grain, 0.6 oz of seafood, 5.4 oz of meat, poultry and eggs, 0.5 oz of nuts and seeds, 1.5 cups of dairy and 33% of total daily energy intake from solid fats and added sugars). Blinding of the menus for participants was achieved by ensuring that the same food components and dishes were used in both diets, from the same core set of foods, so neither diet would distinctly resemble a TAD or DGA based diet. Different compositions of the same core foods were used to match the diet patterns and were designed to be 8-day cyclic menus by the study dietitian. Participants did not have information on the diet assigned, and even while consuming food at the research center (which happened a minimum of twice a week), they were chaperoned by study staff and did not interact with other study participants.

Study diet composites were prepared by pooling and blending (by intervention) and were used in proximate analyses to verify that the designed diet did meet requirements to match the DGA and TAD patterns. Study participants received all foods and drinks to consume for 8 weeks they were enrolled in the intervention. All food and drinks were prepared and packaged in the metabolic kitchen at the WHNRC where the study was conducted. Participants picked up packed meals from the WHNRC, twice a week when they visited the center. They were given specific instructions to refrigerate or freeze appropriate foods till it is time for consumption. Dietary adherence was monitored using checklists that participants turned in for each day during these bi-weekly visits. These checklists were pre-made for each day by study staff, to include a list of foods provided to the participant for each study day, along with space to answer the following questions: ‘time of meal’, ‘% food consumed’, ‘medications consumed’, and ‘notes’ to add any open-ended comments. A sample study checklist is provided in Supplemental Table 1. They were also instructed not to clean their dishes once they consumed each food or drink, and were asked to return containers as is, which were weighed at the WHNRC metabolic kitchen, to evaluate adherence to protocol. Based on both self-reported checklists, weigh backs, as well as 24-h urinary nitrogen measurements (measures taken at week 1, week 4, and week 7 of the intervention), adherence to the provided diet was between 80 and 95%, as reported in depth in the methods manuscript, along with more details about the menu and foods included as well [24].

### Healthy Eating Index (HEI) scores

Habitual dietary intake information of each participant was obtained before the feeding intervention began using the Automated Self-Administered 24-h dietary (ASA24) recall system [25]. Dietary recalls were done four times (since a minimum of three is considered optimal to accurately estimate energy intake [26]) once during in-house training when the participant is oriented into the study, twice unannounced over weekday days, and once unannounced over the weekend. All recalls happened within 4 weeks of when the participant was enrolled in the intervention. Data were averaged across all days of collected recalls, and all participants had at least 2 weekdays and one weekend recalls. The Healthy Eating Index (HEI-2015) components and total scores were calculated per individual across all days of ASA24 recalls, using publicly available statistical codes developed to standardized the calculation of the HEI according to guidelines set by the USDA [27].

## Glycopeptide measurement

### Measured peptides

A total of 17 serum proteins were chosen to be measured using targeted MS analyses. Table 1 provides a list of these peptides, along with a summary of their reported associations with cardiometabolic disease which formed the premise of why they were chosen. Briefly, these peptides have previously been shown to be linked to inflammation, hypertension, dyslipidemia, or dysglycemia. All protein standards were obtained from Sigma-Aldrich (Millipore Sigma- Merck KGaA, St. Louis, Missouri).

**Table 1:** Proteins chosen to be measured, and a brief introduction about their implicated role in cardiometabolic disease

Serum protein	Implicated role in cardiometabolic disease
Angiotensinogen (ANT)	Involved in blood pressure regulation (Corvol P et al. 1984, J.Hypertens Suppl. Dec;2(2):S25-30)
Alpha-2-Heremans Schmid Glycoprotein (A2HSG)	Level of glycosylation indicative of metabolic syndrome (Krishnan S et al. 2017, https://doi.org/10.1038/srep43728)
Kallikrein (KLKB1)	Regulates blood pressure (Sharma JN and Narayanan P 2014, https://doi.org/10.1007/978-3-319-06683-7_2)
Hemopexin (Hemo)	Influences angiotensin responsiveness in humans, indirectly regulating blood pressure (Krikken JA et al., 2013, https://doi.org/10.1097/HJH.0b013e32835c1727)
Kininogen-1 (KNG1)	Deficiency increases salt sensitivity induced increase in blood pressure (Majima M et al. 1993, https://doi.org/10.1161/01.hyp.22.5.705)
Alpha-1-anti-trypsin (A1AT)	Deficiency decreases CVD risk (Fahndrich S et al. 2017, https://doi.org/10.1186/s12931-017-0655-1.), dysfunction associated with ischemic stroke risk (Mahta A et al. 2020, https://doi.org/10.1016/j.jocn.2020.04.074)
Alpha-2-macroglobulin (A2MG)	A2MG intricately linked to balance in inflammatory response (Borth W, 1992, 10.1096/fasebj.6.15.1281457)
Alpha-1-acid glycoprotein (AGP1)	Altered glycosylation involved in anti-inflammatory response (Chavan MM et al., 2005, https://doi.org/10.1093/glycob/cwi067)
Vitronectin (VTNC)	Serum concentrations predictive of metabolic syndrome (Alessi MC et al., 2011, https://doi.org/10.1160/TH11-03-0179)
Ceruloplasmin (Ceru)	Elevated serum concentrations in metabolic syndrome and associated with various CVD risk factors (Kim CH et al., 2002, https://doi.org/10.1053/meta.2002.33348)
Fibronectin (Fib)	Low levels associated with increased risk for coronary heart disease (Zhang et al., 2006, https://doi.org/10.1515/CCLM.2006.008)
Apolipoprotein CIII (ApoCIII)	Lipoprotein associated with hypertriglyceridemia (Ramms B and Gordts PLSM, 2018, https://doi.org/10.1097/MOL.0000000000000502)
Apolipoprotein D (ApoD)	Intricately involved in lipid metabolism in ageing and atherosclerosis (Perdomo G and Dong HH, 2009, https://doi.org/10.18632/aging.100004)
Complement C5	Immune mechanisms in blood pressure regulation, as one of the earliest discovered components of immune system, it plays a role in hypertension (Wenzel UO et al., 2017, https://doi.org/10.1152/ajpheart.00759.2016)
Complement C2	
Complement C4 A and B	
Complement Factor I	

### Sample preparation

10-μL serum samples and glycoprotein standards were reduced with 40 μL 25 mM DTT (Promega, WI) at 60 °C for 50 min, followed by alkylation with 20 μL 90 mM iodoacetamide (IAA) (Millipore Sigma, MO) in the dark for 30 min at room temperature, and 60 μL of 0.067 μg/μL trypsin solution (Promega, WI) was added to the mixture. The tryptic digestion was performed in a water bath at 37 °C for 18 h. After the incubation, the reaction was quenched with 10 μL of 18% formic acid.

### Instrument analysis

Tryptic digested samples were directly characterized using an Agilent 1290 infinity ultrahigh-pressure liquid chromatography (UHPLC) system coupled with an Agilent 6490 triple quadrupole (QQQ) mass spectrometer (Agilent Technology, Santa Clara, CA). 2 μL of the sample was injected, and the analytes were separated on an Agilent Eclipse plus C18 (RRHD 1.8 μm, 2.1 mm × 150 mm) analytical column coupled to an Agilent Eclipse plus C18 (RRHD 1.8 μm, 2.1 mm × 5 mm) guard column. A solution of 3% acetonitrile/97% water (E-pure filtered water) containing 0.1% formic acid and 90% acetonitrile/10% waster containing 0.1% formic acid were used as solvents A and B, respectively (acetonitrile (Honeywell, NJ), formic acid (Fisher Scientific, MA)). The chromatography gradient consisted of 0–20% solvent B over 0–20 min, 20–44% solvent B over 20–47 min, 44–100% solvent B over 47–51 min, and holding at 100% solvent B for 51 min to 64 min. The flow rate was set to 0.5 mL/min. Peptides and glycopeptides were monitored using a dynamic multiple reaction monitoring (dMRM) mode based on the transitions reported as the previous method [28].

### Data processing

The acquired dMRM data were analyzed with Agilent MassHunter Quantitative Analysis B.8.0 software. Signal-to-noise ratio (S/N) of 3 was chosen for the limit of detection, and S/N of 6 was selected as the threshold for the limit of quantitation. Peak areas acquired from the software were used for quantitation. For the standard protein quantitation, the linear curve was determined by evaluating the concentration range where the signal varies linearly with the concentration. The amount of each glycopeptide was quantified as the intensity of each glycopeptide divided by its corresponding unglycosylated peptide.

## Statistical analyses

The sample size and power were based on the primary endpoint fasting insulin and are described elsewhere [4]. Briefly, with 17 participants/group, the study was powered to detect a 5.3-mIU/mL difference in insulin (the primary outcome variable for the study), at a 5% level of significance using a 2-tailed test. This translated to a 0.75 effect size. Adding an attrition rate of 20%, the study sample became 22/group.

### Data analysis

Mol% for sialylated and fucosylated peptides were calculated for each peptide as a ratio of individual sums of mono-, di- or poly- glycosylated peptides to that of the total peptide. Total peptides, individual glycopeptides as well as mol% glycopeptides were used in subsequent analyses [19]. To avoid issues with non-conformity of data to a normal distribution, non-parametric tests were used for uni- and bi-variate analyses, where possible. Several parameters were not able to be transformed to fit the normal distribution assumptions. For univariate analyses, screening for outliers was done using Huber and Cauchy tests, and variables were transformed (Johnson transforms) to address outliers. Mahalanobis distance test was used to verify multivariate normal distribution before use in multivariate statistical analyses.

### Baseline profiling of the glycoproteome parameters

Correlation analyses (Spearman’s rho) were used to evaluate associations between habitual dietary intake, clinical parameters, and the glycoproteome parameters. Further, participants were divided into subgroups based on (a) menopausal status, since type and extent of glycosylation are influenced by this [29] [pre (*n* = 23) vs. post, (*n* = 21)]; (b) based on the number of metabolic syndrome characteristics they had [dyslipidemia (DL—characterized by low HDL-c, high LDL-c, high TG, or high TC; *n* = 18), or dyslipidemia and glucose intolerance (DL + GIT- in addition to dyslipidemia, high fasting glucose, or high 2 h glucose following an oral glucose tolerance test); *n* = 26]; and (c) based on their BMI status (overweight OW; *n* = 15 vs. obese OB; *n* = 29). Non-parametric van der Waerden’s tests were used to compare glycoproteomic parameters between these subgroups, followed by Benjamini–Hochberg multiple comparison corrections.

### Effect of the dietary intervention on the glycoproteome parameters

Differences between fasting data obtained at baseline week (wk0) and following the 8-week intervention exposure (week8) were calculated for all data. Non-parametric van der Waerden’s tests were used to compare changes due to exposure to the diet between DGA and TAD groups, and Benjamini–Hochberg multiple comparison corrections. Transformed (Johnson transformed) ‘change’ data were used in a PLS-DA model to evaluate the discriminatory ability of change in clinical and glycoproteomic parameters to describe the effect of the intervention, while also identifying clinical and glycoproteomic parameters that covary. The PLS-DA model, a variation of the PLS regression, was chosen as opposed to a PCA. The PLS model is supervised (i.e. it can be trained to ‘learn’ the difference between groups) and validated, unlike the PCA, while retaining the ability to derive inference from the covariance matrix of X and Y variables to signal which variables covary, similar to PCA. It is especially useful when there is a greater number of variables than cases (i.e. wide data) and when the variables have a high degree of inter-correlatedness. The PLS-DA model was built to predict Group (DGA vs. TAD) as the dependent variable using change in mol% glycoproteomic, anthropometric, and clinical measures as independent variables. Leave-one-out cross-validation was used since the sample size was small, this was not computationally intensive, and this is better than hold-back since it has less bias in the regression coefficients [30] to verify the validity of the built models. Q2 (goodness of prediction) and R2 (coefficient of multiple determination) values were used to evaluate the goodness of fit. Independent variables that were important contributors to differentiating between the groups—DGA and TAD, were identified using a variable importance plot (VIP) with a VIP cut-off score of > 1. All data analyses were done in R (version 3.6.0) and JMP Pro 14.1 (SAS Institute, Cary NC).

## Results

### Associations between baseline clinical characteristics and glycoproteome

**Fig. 1:**
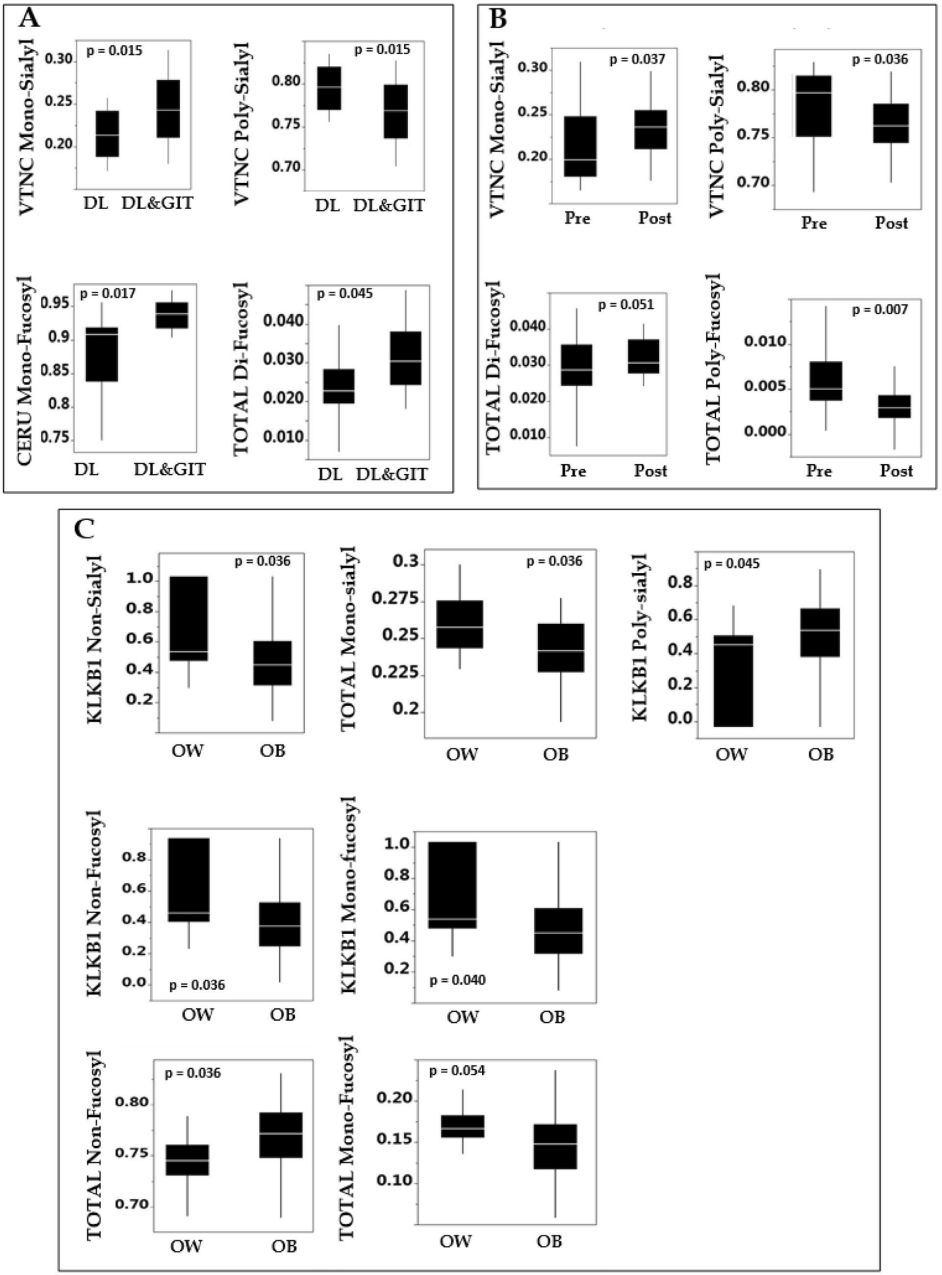
All significantly different glycovariant mol% of individual or total peptides arranged within each panel from left to right in increasing order of glycosylation (none-mono-di-poly). Box and whisker plots showing the median ± interquartile range values, with p-values inset. Panel A shows differences based on screening characteristics—DL = dyslipidemic (*n* = 18), DL + GIT (*n* = 26) = dyslipidemic + glucose intolerant. Panel B presents differences between pre (*n* = 23- and post-menopausal women (*n* = 21). Panel C shows differences between overweight (OW—BMI between 25 and 30 kg/m^2^, *n* = 15) and obese (OB, BMI between 30 and 40 kg/m^2^, *n* = 29) individuals. *VTNC* Vitronectin, *CERU* ceruloplasmin, *TOTAL* all peptides together, *KLKB1* Kallikrein, *sialyl*—sialylated, *fucosyl* fucosylated

The glycovariants that are significantly different at week 0 in the 44 participants separated into subgroups based on their metabolic syndrome status, menopausal status, and BMI status are depicted in Fig. 1 in panels A, B, and C. VTNC mono-sialylated proteins were higher in women with both dyslipidemia (DL) and glucose intolerance (GIT) (Panel A) compared to women with only DL (*p* = 0.015), while poly-sialylated proteins were higher in women with DL alone compared to both DL + GIT (*p* = 0.015). Ceru mono-fucosylated peptides were in greater abundance in women with DL + GIT compared to women with DL alone (*p* = 0.017). Total di-fucosylated proteins were also higher in DL + GIT compared to women with DL alone (*p* = 0.045). Based on these, an overall profile of higher sialylation being associated with having fewer metabolic syndrome characteristics emerges.

Vitronectin mono-sialylated proteins were higher in postmenopausal women (see Fig. 1 Panel B) compared to premenopausal women (*p* = 0.037), while poly-sialylated proteins were lower (*p* = 0.036). Total poly-fucosylated proteins were higher in pre-menopausal women compared to postmenopausal women (*p* = 0.007), while there was a trend toward total di-fucosylated proteins being higher in postmenopausal women compared to premenopausal women (*p* = 0.051).

Non-sialylated KLKB1 was higher in overweight women (see Fig. 1 Panel B) compared to obese (*p* = 0.036), as were mono-fucosylated KLKB1 (*p* = 0.040), non-fucosylated KLKB1 (*p* = 0.040), and total mono-sialylated proteins (*p* = 0.036). On the contrary, poly-sialylated KLKB1 was higher in obese compared to overweight women (*p* = 0.045), as were total non-fucosylated proteins (0.034), and total mono-fucosylated were higher in overweight women (0.054).

### Associations between baseline habitual diet and glycoproteome

**Fig. 2:**
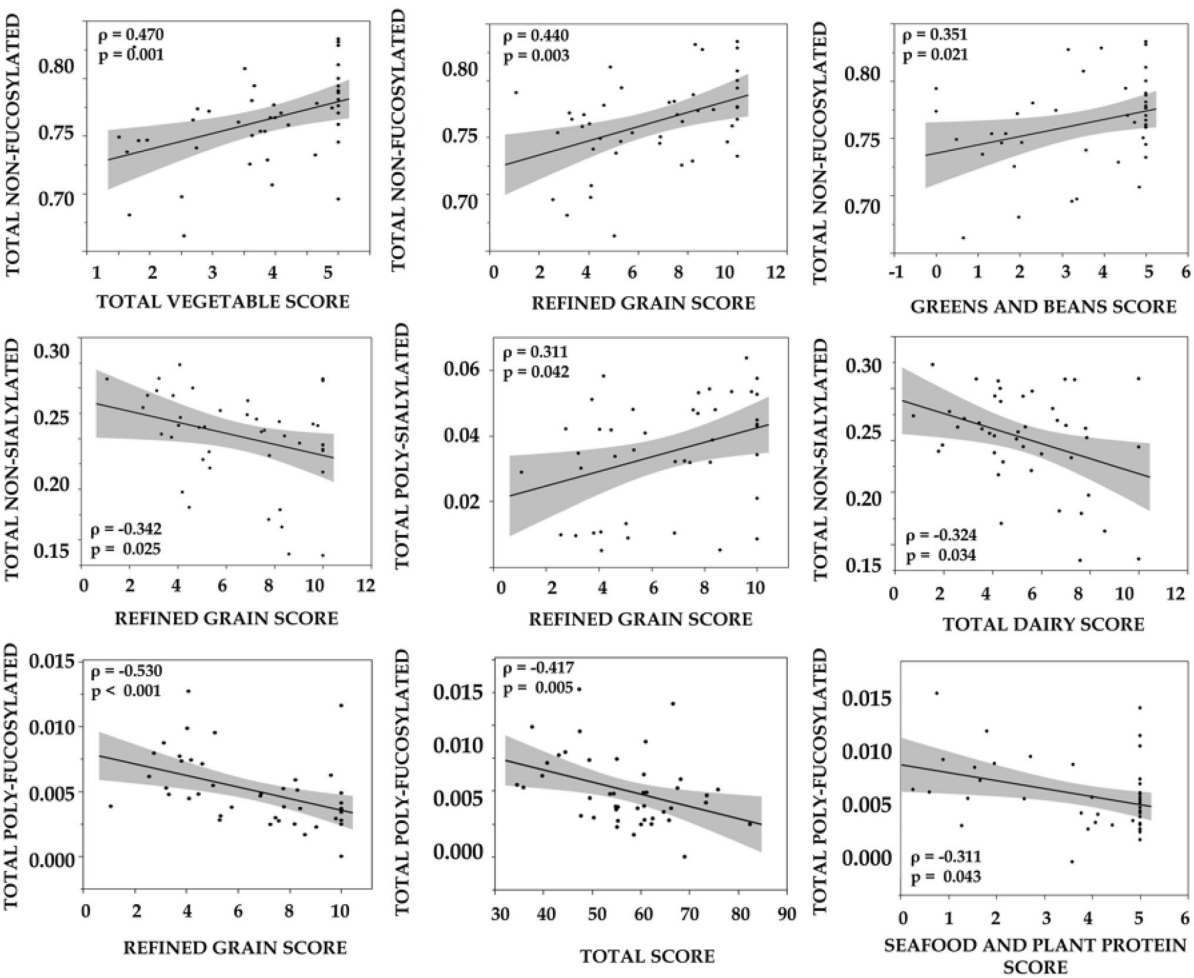
Correlation based significant associations between glycovariant mol% (*y*-axis) and HEI sub-category scores (*x*-axis) with inset Spearman’s rho (*ρ*) and *p* values. For total vegetables, greens and beans, seafood and plant proteins, total dairy and total score higher score reflects both higher intake of these food groups and a ‘healthy’ diet. For refined grain a higher score indicates lower intake and a ‘healthy’ diet

Correlation analyses at baseline revealed associations between baseline diet quality indices and glycovariant mol% (see Fig. 2 and Supplemental Table 2). Total non-fucosylated glycovariant proteins were positively associated with sub-scores representing ‘total vegetable’, ‘greens and beans’, and ‘refined grain’ intake. Total poly-fucosylated glycovariant mol% was inversely associated with ‘total score’ (healthy eating index, HEI), ‘refined grain’, and ‘seafood and plant protein’ intake. Total non-sialylated proteins were inversely associated with ‘total dairy’ and ‘refined grain’ while total poly-sialylated proteins were positively associated with ‘refined grain’ score. A healthy diet pattern that includes more vegetables, more seafood, and reduced refined grain appears to be associated with greater poly-sialylated proteins, while higher fucosylation appears to be associated with a less healthy diet.

### Change in glycoproteome related to controlled diet intervention

**Fig. 3:**
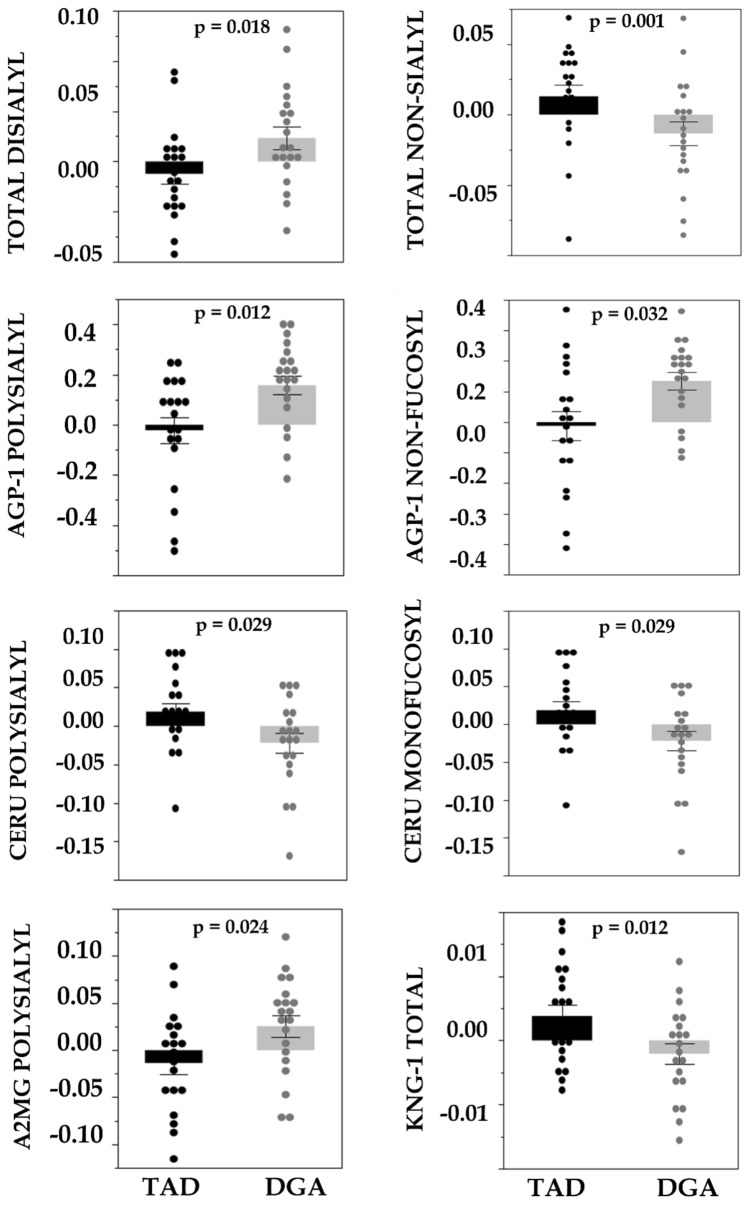
Mol% glycoprotein changes (wk8–wk0) comparing DGA and TAD groups. Box plot represents median + IQR, and points show data (there were no statistical outliers) with p values inset. Only analytes with significant diet differences (*p* < 0.05) are depicted here. *A2MG* alpha-2-macroglobulin, *KNG-1* Kininogen, *CERU* ceruloplasmin, *AGP-1* alpha-1-acid glycoprotein, *sialyl* sialylated, *fucosyl* fucosylated, *TAD* typical American diet, *DGA* Dietary guidelines for American diet

The change in total kininogen concentration was more positive (Fig. 3) in TAD compared to the DGA group. Change in total di-sialylated proteins was higher in DGA compared to the TAD group, as was poly-sialylated A2MG protein, poly-sialylated AGP-1, and non-fucosylated AGP-1. Mol% poly-sialylated Ceru protein change was more positive in TAD compared to DGA, similar to Ceru mono-fucosylated. Mol% total non-sialylated protein change was higher in TAD compared to the DGA group.

**Fig. 4:**
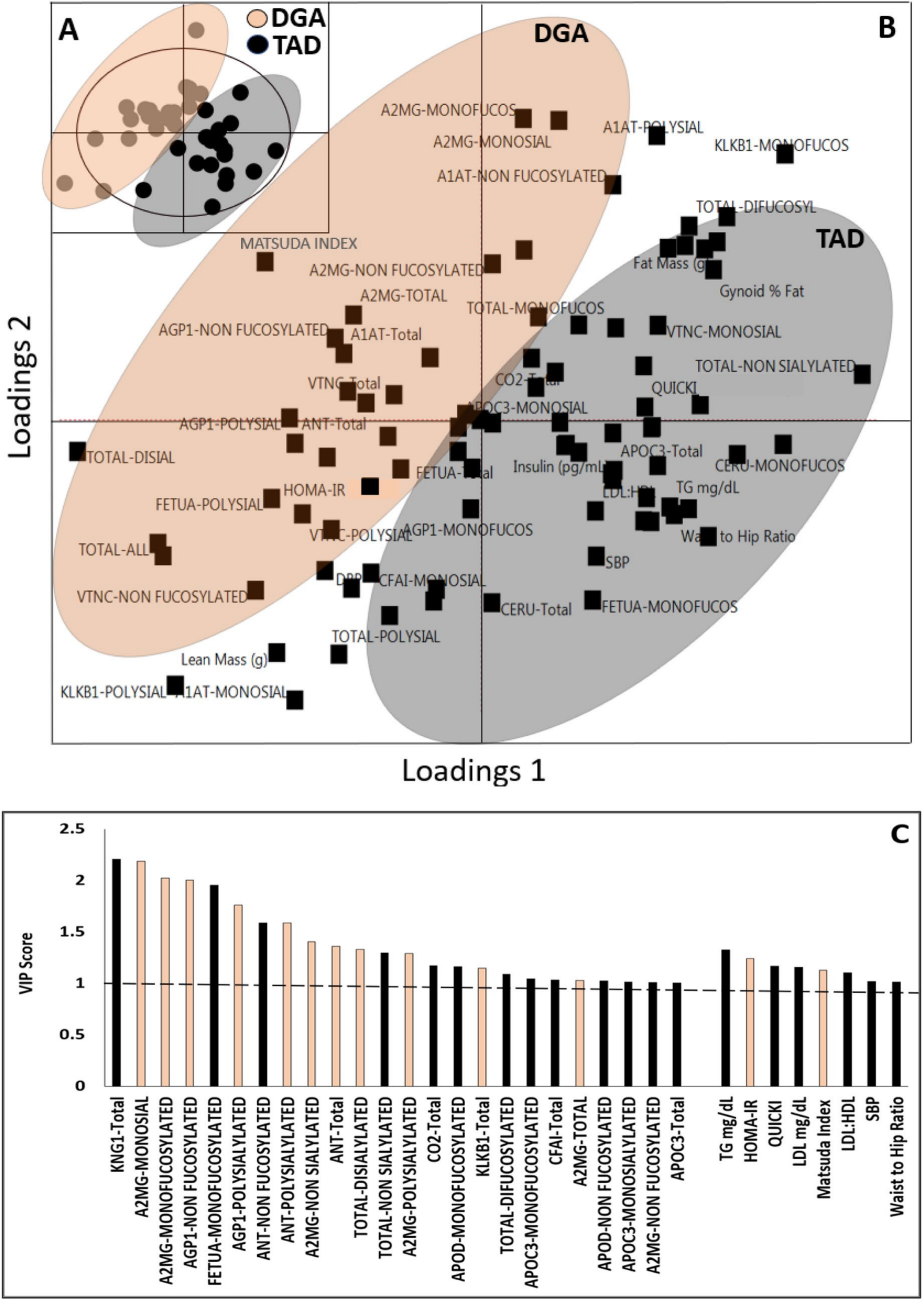
Loadings and scores plot of a PLS-DA model generated to predict ‘Group’ using difference in wk8–wk0 in anthropometric, clinical and glycovariant data. The scores plot (**a**) shows the participant distribution across the n-dimensions is inset within the loadings plot (**b**) which shows the variables (dimensions). In the scores plot (**a**) the black dots represent scores from subjects fed the TAD and orange dots represent scores of participants fed the DGA. In both scores and loadings plot (**b**), the orange highlight ellipses represent DGA and dark grey ellipses highlight TAD group. **c** Displays the VIP variables with VIP score > 1, which significantly contribute to the model discrimination of DGA and TAD groups, coded with orange for variables that are associated with change in DGA and black for TAD

The PLS-DA model based on the change in clinical, anthropometric, and glycoprotein variables is depicted in Fig. 4. The PLS-regression represents 69% (*R*^2^*X*) of the variance in the *X* variables (independent variables) and 85% (*R*^2^*Y*) of the variance in the *Y* variable (Group—DGA vs TAD). The Q2 was 0.80, and the model had 32 variables that had a VIP score of > 1. The scores plot shows the separation of the groups (DGA = orange, TAD = grey). The corresponding loadings plot, when considered along with the VIP plot suggests that the TAD group (grey) is characterized by colinear changes in TG, SBP, QUICKI, LDL-c, LDL: HDL ratio, waist-hip ratio, KNG-1, CFAI, ApoC3, mono-fucosylated—Fetuin, ApoD, ApoC3, mono-sialylated Apo C3, non-fucosylated—ANT, ApoD, A2MG, non-sialylated total proteins, and di-fucosylated total proteins. The DGA group (orange) was characterized by colinear changes in HOMA-IR, Matsuda index, total A2MG, total ANT, total di-sialylated proteins, poly-sialylated—AGP1, ANT, A2MG, di-sialylated—total proteins, non-fucosylated—AGP1, mono-sialylated -A2MG, mono-fucosylated—A2MG, and non-sialylated—A2MG. Overall, more glycoproteomic variables were involved in explaining the difference between DGA and TAD compared to clinical parameters.

## Discussion

To our knowledge, this is the first time the effect of diet on the serum glycoproteome profile has been evaluated, both in the form of association with habitual diet and as a result of change induced by a controlled feeding intervention. Women with a greater number of metabolic syndrome characteristics had greater fucosylation of proteins. Further, a lower refined grain intake was associated with higher sialylation of proteins and reduced fucosylation. The DGA diet increased total di-sialylated proteins, poly-sialylated A2MG, and AGP1, and non-fucosylated AGP1. The TAD group, on the other hand, showed increased KNG1, and poly-sialylated and mono-fucosylated Ceru. Our multivariate analyses revealed change in di- and poly-sialylated and non-fucosylated proteins, HOMA-IR and Matsuda index, to carry the most group discriminating information among variables in the DGA group. In the TAD group, discriminators were: change in systolic blood pressure, QUICKI, LDLc, TG, KNG-1, and mostly non-fucosylated, mono-fucosylated, and non-sialylated proteins- apoC3, CFAI, and ApoD. Even though a previous study reported differences in glycosylation based on menopausal status [29], we did not see any differences. BMI categories also did not display clear differences in the pattern of glycosylation in this study. Based on this, an overall profile of the DGA and healthier diets being associated with increased sialylation; and less nutrient-dense diets being associated with reduced sialylation/increased fucosylation emerges.

### Greater total sialylation and healthier diet pattern

In the current study, there was a positive association between poly-sialylated proteins and consuming a healthier diet based on the HEI scores. Also, there was an increase in total di-sialylated protein in the DGA group and a decrease of the same in the TAD group. Studies have shown that sialic acid can either mask binding sites or make binding sites available to receptor ligands [31]. Sialylation is specifically noted for its effects on protein half-life, clearance, and functionality of proteins, wherein loss of sialic acid is associated with reduced half-life and functionality [32]. Polysialic acid in particular has been shown to modulate cellular interactions of dendritic cells and other agents of innate immune activation [33], which is intricately linked with the development of type 2 diabetes [34], and cardiovascular mortality in individuals with type 2 diabetes [35]. It is important to note here that while there are studies that show that higher circulating sialic acid concentration is linked to type 2 diabetes, and its downstream vascular pathologies [36], the current report focuses on sialic acids bound to proteins, which is a different measure. Based on our current report, it appears that the DGA diet pattern or a nutrient-dense diet as indicated by HEI scores is associated with higher sialylation, and reduced fucosylation.

A primary difference between DGA and TAD was in their dietary fiber (soluble and insoluble) content: the DGA diet had ~ 29 g/day of dietary fiber, while the TAD had ~ 19 g/day. Dietary fiber, upon fermentation in the large intestine, produces short-chain fatty acids (SCFAs—acetate, butyrate, and propionate), which can influence several physiological functions [37]. Expression of enzymes involved in glycosylation (glycosidases, transferases) can be up or downregulated by epigenetic modifications—acetylation, phosphorylation, or methylation [38], and this, in turn, influences the glycosylation process. Butyrate, a gut microbiota-derived SCFA [39] is a known nutritionally derived epigenetic modifier since it is a histone deacetylase inhibitor [40]. Butyrate alters erythropoietin glycosylation in Chinese hamster ovary cells in vitro [41], and more specifically increased expression of α 2,6-sialyltransferase resulting in greater sialylation of proteins [42–44]. Dietary fat [which was also different between DGA (26% of daily energy) and TAD (34% of daily energy)] has also been shown to alter glycosylation pattern in duodenal cells in mice [45], by altering the gut microbiota; however, whether this translates to systemic change in glycosylation is not yet clear. These are likely mechanistic links by which the diet based on DGA altered the sialylation of serum proteins. While it is outside the scope of the current study to evaluate these mechanisms, future studies should investigate this further.

### Individual glycoprotein changes due to intervention

Total KNG1 was elevated in TAD as a result of the intervention, but not in the DGA group. KNG1, when activated by KBKB1, has vasodilator and diuretic effects, and knockouts of KNG1 result in increased blood pressure responses to salt loading [46]. Bovine kininogen and bradykinin have been shown to act as vasodilators, and are considered biologically active proteins and of interest in the nutraceutical industry [47]. Upon a closer inspection, we observed an inverse association between baseline dairy consumption (dairy HEI) and total KNG1 (*r* = − 0.342, *p* = 0.025). Our DGA group went from having a 4.8/10 HEI score for dairy at baseline to a 10/10 on the intervention, while the TAD group went from a 5.89/10 score at baseline to a 5/10. Based on this, one might speculate that the reduction in dairy in the TAD group resulted in fewer functional kinins from the diet, making it necessary for it to be produced endogenously. This suggests that this increase in KNG1 was necessary for maintaining blood pressure, which it did in the TAD group since there was no change in systolic or diastolic blood pressure. However, this hypothesis needs further verification.

In the current study di-sialylated glycoproteins (AGP1 and A2MG) increased in DGA, but not in the TAD group. AGP1 is an acute-phase protein, whose biological role is varied, including binding to leptin receptors to influence the energy homeostasis regulatory pathway [48] and as an anti-inflammatory [49] and anti-platelet aggregating factor [50]. AGP1 glycosylation level and type have been documented to change in disease states [51]. There was a reciprocal increase in sialylation and reduction in fucosylation by 60% in both AGP1 and A2MG when acute phase response was triggered in rats [52]. In the current study, the increase in sialylation of total measured AGP1 suggests a greater inhibition of platelet aggregation, which is cardioprotective [53]. A2MG is an inhibitor of proteinase activity, it inhibits fibrinolysis by inhibiting kallikrein and plasmin, and inhibits coagulation by inhibiting thrombin [54]. It is also a potent anti-inflammatory agent [55]. Aging is generally associated with more pro-inflammatory glycans that are less sialylated [56], and the DGA intervention increasing sialylation of A2MG can be indicative of reduced inflammation.

Our PLS-DA models indicated covarying HOMA-IR, Matsuda index, and di-, poly and non-fucosylated proteins. While this agrees with the general trend that the DGA diet had increased sialylation, the covarying HOMA-IR and Matsuda index is counter-intuitive. Increasing HOMA-IR values indicate insulin resistance, and increasing Matsuda index values indicate insulin sensitivity. The fact that change in these concurrently occur with increased sialylation and reduced fucosylation suggests the difference in the mechanism by which the glycovariants affect metabolic health. HOMA-IR is a function of fasting plasma insulin and glucose, while the Matsuda index is calculated as a function of fasting and post-prandial (following an OGTT) plasma insulin and glucose. How increased sialylation and reduced fucosylation relates to these different surrogates for insulin effectiveness at the systemic level remains unclear and requires further evaluation. A recent study identified reduced fucosylation and higher sialylation in individuals with type 2 diabetes. Further, they reported that it is not the total sialylation, but the type of sialyl-linkage (alpha-2,3 vs alpha-2,6 glucosidic bond) that affects the function of the peptide [57]. So, how sialylation, which appears to be increasing with the DGA diet in the current study, affects risk for type 2 diabetes needs further investigation.

## Strengths and limitations

Of the total 17 proteins, we only found significant changes in four. This could be because of the small sample size, or the short duration (8wk) of the study. However, the only previous study evaluating the DGA pattern was done for 4wk and reported minimal changes in their clinical outcomes. Our study was the first-ever 8wk intervention to evaluate the DGA diet pattern. A longer duration intervention may show greater changes. Alternately, even if the diet had an effect, it could either be small or highly variable and may affect each protein differently in different individuals, and these will need both larger sample sizes and longer duration interventions to evaluate. Yet another limitation is that these are secondary analyses from a clinical trial, and the primary study was powered to detect changes in fasting insulin concentrations. This indicates that there is likely inflation of type I error. In the current study, a false-positive rate correction was applied to ensure that this is being addressed, adding robustness to our findings. The use of block randomization leaves the study vulnerable to selection bias, since the treatment that has not been randomized frequently so far in unmasked groups is more likely to be chosen next [58]. While our PLS-DA model was used to draw inference and not in a predictive capacity, and was validated internally, future studies with larger sample sizes and comparing healthy vs. diseased populations are needed to verify the findings from this current report. A strength of the current study was that the observations noticed in baseline data (higher sialylation associated with a healthy diet, higher fucosylation with a less healthy diet) were largely aligned with what was observed as a result of a controlled feeding intervention (greater sialylation following the DGA diet) with one exception.

## Conclusions and future direction

A majority of mammalian proteins are glycosylated, and these processes play important roles in protein function. The results of this study suggest that dietary patterns can affect post-translational modification, specifically *N*-glycosylation. The current study is the first to show this relationship in humans. Given the association between diet and glycan composition of proteins we report here, it is important to investigate if the serum glycoproteome can be used to identify biomarkers indicative of dietary patterns. A clinical intervention trial comparing diet patterns associated with health and disease can be used to evaluate the circulating glycoproteome, along with changes in SCFAs and the gut microbiome. This would test our proposed mechanism, and verify findings currently being reported. One approach to precision nutrition is to include nutritional physiology and biochemistry knowledge in a systems biology framework and to evaluate inter-individual variability through the application of comprehensive phenotyping tools. In this regard, the glycoproteome is an important addition to the armamentarium.

## Supplementary Information

Below is the link to the electronic supplementary material.

Supplementary file1 (DOCX 147 kb)

Supplementary file2 (DOCX 25 kb)

## Abbreviations

PTM: Post-translational modification
DGA: Dietary Guidelines for Americans
TAD: Typical American Diet
UHPLC-MS: Ultra-high performance liquid chromatography-mass spectrometry
ASA24: Automated self-administered 24 h dietary recall system
HEI: Healthy eating index
ApoD: Apolipoprotein D
ApoCIII: Apolipoprotein CIII
ANT: Angiotensinogen
A2MG: Alpha-2-macroglobulin
A1AT: Alpha-1-antitrypsin
AGP1: Alpha-1-acid glycoprotein/orosomucoid
A2HSG: Alpha-2-Heremans Schmid Glycoprotein
CFAI: Complement Factor I
Ceru: Ceruloplasmin
Fib: Fibronectin
Hemo: Hemopexin
KLKB1: Kallikrein
KNG1: Kininogen
VTNC: Vitronectin

## References

[1] USDA (2015). Dietary Guidelines for Americans 2015, 8th.

[2] Minister of Agriculture and Agri-Food Canada (2013). Best practices for food-based clinical trials: guidance for planning, conducting and reporting on human studies to support health claims.

[3] Schroeder N, Park YH, Kang MS, Kim Y, Ha GK, Kim HR, Yates AA, Caballero B (2015). A randomized trial on the effects of 2010 Dietary Guidelines for Americans and Korean diet patterns on cardiovascular risk factors in overweight and obese adults. J Acad Nutr Diet.

[4] Krishnan S, Adams SH, Allen LH, Laugero KD, Newman JW, Stephensen CB, Burnett DJ, Witbracht M, Welch LC, Que ES, Keim NL (2018). A randomized controlled-feeding trial based on the Dietary Guidelines for Americans on cardiometabolic health indexes. Am J Clin Nutr.

[5] Playdon MC, Moore SC, Derkach A, Reedy J, Subar AF, Sampson JN, Albanes D, Gu F, Kontto J, Lassale C, Liao LM, Männistö S, Mondul AM, Weinstein SJ, Irwin ML, Mayne ST, Stolzenberg-Solomon R (2017). Identifying biomarkers of dietary patterns by using metabolomics. Am J Clin Nutr.

[6] Tebani A, Bekri S (2019). Paving the way to precision nutrition through metabolomics. Front Nutr.

[7] Brennan L (2013). Metabolomics in nutrition research: current status and perspectives. Biochem Soc Trans.

[8] Reily C, Stewart TJ, Renfrow MB, Novak J (2019). Glycosylation in health and disease. Nat Rev Nephrol.

[9] Pitti T, Chen CT, Lin HN, Choong WK, Hsu WL, Sung TY (2019). N-GlyDE: a two-stage N-linked glycosylation site prediction incorporating gapped dipeptides and pattern-based encoding. Sci Rep.

[10] Mitra N, Sinha S, Ramya TN, Surolia A (2006). N-linked oligosaccharides as outfitters for glycoprotein folding, form and function. Trends Biochem Sci.

[11] Ohtsubo K, Marth JD (2006). Glycosylation in cellular mechanisms of health and disease. Cell.

[12] Shental-Bechor D, Levy Y (2008). Effect of glycosylation on protein folding: a close look at thermodynamic stabilization. Proc Natl Acad Sci USA.

[13] Mukherjee A, Morales-Scheihing D, Butler PC, Soto C (2015). Type 2 diabetes as a protein misfolding disease. Trends Mol Med.

[14] Rudman N, Gornik O, Lauc G (2019). Altered N-glycosylation profiles as potential biomarkers and drug targets in diabetes. FEBS Lett.

[15] Varki A, Cummings RD, Esko JD, Stanley P, Hart GW, Aebi M, Darvill AG, Kinoshita T, Packer NH, Prestegard JH, Schnaar RL, Seeberger PH (2015) Essentials of glycobiology. In. NBK453074 27010055

[16] Keeley TS, Yang S, Lau E (2019). The diverse contributions of fucose linkages in cancer. Cancers (Basel).

[17] Varki A, Gagneux P (2012). Multifarious roles of sialic acids in immunity. Ann N Y Acad Sci.

[18] Krishnan S, Huang J, Lee H, Guerrero A, Berglund L, Anuurad E, Lebrilla CB, Zivkovic AM (2015). Combined high-density lipoprotein proteomic and glycomic profiles in patients at risk for coronary artery disease. J Proteome Res.

[19] Krishnan S, Shimoda M, Sacchi R, Kailemia MJ, Luxardi G, Kaysen GA, Parikh AN, Ngassam VN, Johansen K, Chertow GM, Grimes B, Smilowitz JT, Maverakis E, Lebrilla CB, Zivkovic AM (2017). HDL Glycoprotein composition and site-specific glycosylation differentiates between clinical groups and affects IL-6 secretion in lipopolysaccharide-stimulated monocytes. Sci Rep.

[20] Bentley-Lewis R, Koruda K, Seely EW (2007). The metabolic syndrome in women. Nat Clin Pract Endocrinol Metab.

[21] Rochlani Y, Pothineni NV, Mehta JL (2015). Metabolic syndrome: does it differ between women and men?. Cardiovasc Drugs Ther.

[22] Barnard RJ (1991). Effects of life-style modification on serum lipids. Arch Intern Med.

[23] Hirst JA, Stevens RJ, Farmer AJ (2014). Changes in HbA1c level over a 12-week follow-up in patients with type 2 diabetes following a medication change. PLoS ONE.

[24] Krishnan S, Lee F, Burnett DJ, Kan A, Bonnel EL, Allen LH, Adams SH, Keim NL (2020). Challenges in designing and delivering diets and assessing adherence: a randomized controlled trial evaluating the 2010 dietary guidelines for Americans. Curr Dev Nutr.

[25] Subar AF, Kirkpatrick SI, Mittl B, Zimmerman TP, Thompson FE, Bingley C, Willis G, Islam NG, Baranowski T, McNutt S, Potischman N (2012). The Automated Self-Administered 24-hour dietary recall (ASA24): a resource for researchers, clinicians, and educators from the National Cancer Institute. J Acad Nutr Diet.

[26] Ma Y, Olendzki BC, Pagoto SL, Hurley TG, Magner RP, Ockene IS, Schneider KL, Merriam PA, Hébert JR (2009). Number of 24-hour diet recalls needed to estimate energy intake. Ann Epidemiol.

[27] National Cancer Institute (2020) Healthy Eating Index: Choosing a method and SAS code website. https://epi.grants.cancer.gov/hei/tools.html. Accessed 6 June 2020

[28] Li Q, Kailemia MJ, Merleev AA, Xu G, Serie D, Danan LM, Haj FG, Maverakis E, Lebrilla CB (2019). Site-specific glycosylation quantitation of 50 serum glycoproteins enhanced by predictive glycopeptidomics for improved disease biomarker discovery. Anal Chem.

[29] Knezevic A, Gornik O, Polasek O, Pucic M, Redzic I, Novokmet M, Rudd PM, Wright AF, Campbell H, Rudan I, Lauc G (2010). Effects of aging, body mass index, plasma lipid profiles, and smoking on human plasma N-glycans. Glycobiology.

[30] Ramyaa R, Hosseini O, Krishnan GP, Krishnan S (2019). Phenotyping women based on dietary macronutrients, physical activity, and body weight using machine learning tools. Nutrients.

[31] Varki A, Cummings RD, Esko JD, Freeze HH, Stanley P, Bertozzi CR, Hart GW, Etzler ME (2009) Essentials of glycobiology. In: NBK1920 20301239

[32] Bork K, Horstkorte R, Weidemann W (2009). Increasing the sialylation of therapeutic glycoproteins: the potential of the sialic acid biosynthetic pathway. J Pharm Sci.

[33] Bax M, van Vliet SJ, Litjens M, García-Vallejo JJ, van Kooyk Y (2009). Interaction of polysialic acid with CCL21 regulates the migratory capacity of human dendritic cells. PLoS ONE.

[34] Pickup JC (2004). Inflammation and activated innate immunity in the pathogenesis of type 2 diabetes. Diabetes Care.

[35] Pickup JC, Mattock MB (2003). Activation of the innate immune system as a predictor of cardiovascular mortality in Type 2 diabetes mellitus. Diabet Med.

[36] Kumar JA, Rai S, Shetty SK, Rai T, Shrinidhi BM, Md S (2013). Predictive value of serum sialic Acid in type-2 diabetes mellitus and its complication (nephropathy). J Clin Diagn Res.

[37] Dalile B, Van Oudenhove L, Vervliet B, Verbeke K (2019). The role of short-chain fatty acids in microbiota-gut-brain communication. Nat Rev Gastroenterol Hepatol.

[38] Lauc G, Vojta A, Zoldoš V (2014). Epigenetic regulation of glycosylation is the quantum mechanics of biology. Biochim Biophys Acta.

[39] Liu H, Wang J, He T, Becker S, Zhang G, Li D, Ma X (2018). Butyrate: a double-edged sword for health?. Adv Nutr.

[40] Berni Canani R, Di Costanzo M, Leone L (2012). The epigenetic effects of butyrate: potential therapeutic implications for clinical practice. Clin Epigenet.

[41] Chung B. JY, Choi O., Kim J (ed) (2001) Effect of sodium butyrate on glycosylation of recombinant erythropoietin, vol 1. Animal cell technology: from target to market. ESACT Proceedings. Springer, Dordrecht. https://doi.org/10.1007/978-94-010-0369-8_46

[42] Yin B, Wang Q, Chung CY, Bhattacharya R, Ren X, Tang J, Yarema KJ, Betenbaugh MJ (2017). A novel sugar analog enhances sialic acid production and biotherapeutic sialylation in CHO cells. Biotechnol Bioeng.

[43] Santell L, Ryll T, Etcheverry T, Santoris M, Dutina G, Wang A, Gunson J, Warner TG (1999). Aberrant metabolic sialylation of recombinant proteins expressed in Chinese hamster ovary cells in high productivity cultures. Biochem Biophys Res Commun.

[44] Lamotte D, Buckberry L, Monaco L, Soria M, Jenkins N, Engasser JM, Marc A (1999). Na-butyrate increases the production and alpha2,6-sialylation of recombinant interferon-gamma expressed by alpha2,6- sialyltransferase engineered CHO cells. Cytotechnology.

[45] Mastrodonato M, Calamita G, Mentino D, Scillitani G (2020). High-fat diet alters the glycosylation patterns of duodenal mucins in a murine model. J Histochem Cytochem.

[46] Gu D, Zhao Q, Kelly TN, Hixson JE, Rao DC, Cao J, Chen J, Li J, Ji X, Hu D, Wang X, Liu DP, He J (2012). The role of the kallikrein-kinin system genes in the salt sensitivity of blood pressure: the GenSalt Study. Am J Epidemiol.

[47] O'Mahony JA, Fox PF (2014) Chapter 2—milk: an overview. In: MPSe, Food FEt (eds) Food science and technology, pp 19–73. https://doi.org/10.1016/B978-0-12-405171-3.00002-7

[48] Sun Y, Yang Y, Qin Z, Cai J, Guo X, Tang Y, Wan J, Su DF, Liu X (2016). The acute-phase protein orosomucoid regulates food intake and energy homeostasis via leptin receptor signaling pathway. Diabetes.

[49] Williams JP, Weiser MR, Pechet TT, Kobzik L, Moore FD, Hechtman HB (1997). alpha 1-Acid glycoprotein reduces local and remote injuries after intestinal ischemia in the rat. Am J Physiol.

[50] Hochepied T, Berger FG, Baumann H, Libert C (2003). Alpha(1)-acid glycoprotein: an acute phase protein with inflammatory and immunomodulating properties. Cytokine Growth Factor Rev.

[51] Ceciliani F, Pocacqua V (2007). The acute phase protein alpha1-acid glycoprotein: a model for altered glycosylation during diseases. Curr Protein Pept Sci.

[52] Chavan MM, Kawle PD, Mehta NG (2005). Increased sialylation and defucosylation of plasma proteins are early events in the acute phase response. Glycobiology.

[53] Clappers N, Brouwer MA, Verheugt FW (2007). Antiplatelet treatment for coronary heart disease. Heart.

[54] Borth W (1992). Alpha 2-macroglobulin, a multifunctional binding protein with targeting characteristics. FASEB J.

[55] Vandevyver S, Dejager L, Vandenbroucke RE, Libert C (2014). An acute phase protein ready to go therapeutic for sepsis. EMBO Mol Med.

[56] Merleev AA, Park D, Xie Y, Kailemia MJ, Xu G, Ruhaak LR, Kim K, Hong Q, Li Q, Patel F, Wan YY, Marusina AI, Adamopoulos IE, Lal NN, Mitra A, Le ST, Shimoda M, Luxardi G, Lebrilla CB, Maverakis E (2020). A site-specific map of the human plasma glycome and its age and gender-associated alterations. Sci Rep.

[57] Dotz V, Lemmers RFH, Reiding KR, Hipgrave Ederveen AL, Lieverse AG, Mulder MT, Sijbrands EJG, Wuhrer M (1862). van Hoek M (2018) Plasma protein N-glycan signatures of type 2 diabetes. Biochim Biophys Acta Gen Subj.

[58] Efird J (2011). Blocked randomization with randomly selected block sizes. Int J Environ Res Public Health.

