# Supplementary material for: Diet affects glycosylation of serum proteins in women at risk for cardiometabolic disease
Source: Eur J Nutr. 2021 Mar 26;60(7):3727–41. doi: 10.1007/s00394-021-02539-7 (PMC8437848; doi:10.1007/s00394-021-02539-7)
Supplement: Supplementary file 1 — Supplementary file1 (DOCX 147 kb) [file 394_2021_2539_MOESM1_ESM.docx]

**
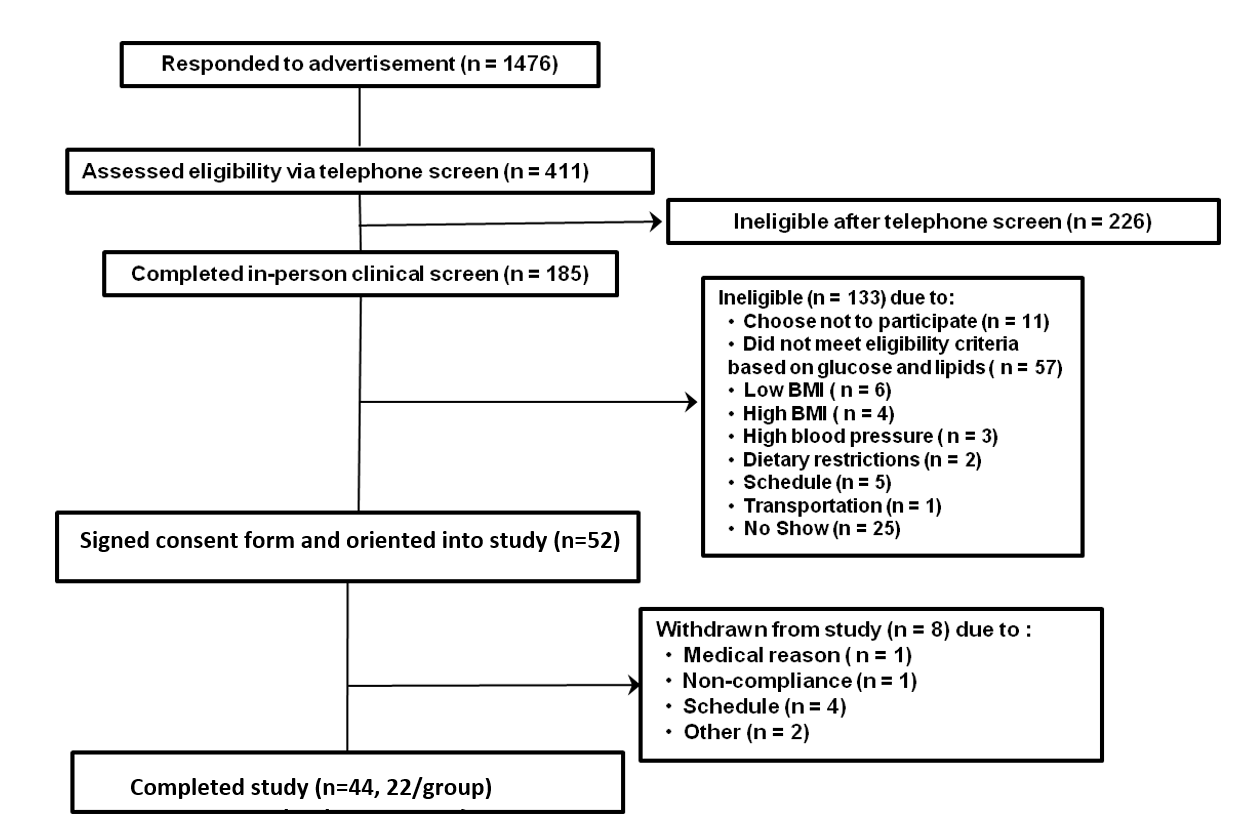
**

**Supplemental Figure 1:** CONSORT diagram representing the volunteers who were screened, consented, and completed the study. CONSORT, Consolidated Standards. In the current study completed study volunteers were used in analysis.
