# Supplementary material for: Diet affects glycosylation of serum proteins in women at risk for cardiometabolic disease
Source: Eur J Nutr. 2021 Mar 26;60(7):3727–41. doi: 10.1007/s00394-021-02539-7 (PMC8437848; doi:10.1007/s00394-021-02539-7)
Supplement: Supplementary file 2 — Supplementary file2 (DOCX 25 kb) [file 394_2021_2539_MOESM2_ESM.docx]

**Supplemental table 1.** Sample checklist

[Front]

| **Metabolic kitchen phone number** | **Participant ID:** | | |
| --- | --- | --- | --- |
| **Dietitian’s email** | **Date:** | | |
| **---Breakfast---** | | **Eaten** | **Time** |
| Cream (D3/S/BLUE) | |  |  |
| Sugar (D3/S/BLUE) | |  |  |
| Bagel with apricot spread (D3/S/BLUE) | |  |  |
| Egg scramble with spicy ketchup (D3/S/BLUE) | |  |  |
| Breakfast Potatoes with spicy ketchup (D3/S/BLUE) | |  |  |
| Orange Drink (D3/S/BLUE) | |  |  |
| **---lunch---** | | **Eaten** | **Time** |
| Barbeque beef sandwich (D3/S/BLUE) | |  |  |
| Raspberry soda (Add sparkling water to syrup) (D3/S/BLUE) | |  |  |
| **---Dinner---** | | **Eaten** | **Time** |
| Teriyaki Veggies with Chicken and Rice (D3/S/BLUE) | |  |  |
| Cran-Raspberry Drink (D3/S/BLUE) | |  |  |
| **---Snacks---** | | **Eaten** | **Time** |
| Banana Chips (D3/S/BLUE) | |  |  |
| Peanut Butter (D3/S/BLUE) | |  |  |
| Cheesecake Pudding with Dried Cherries (D3/S/BLUE) | |  |  |
| Iced Tea, Sweet (D3/S/BLUE) | |  |  |
| How many of the provided **coffee/tea (circle which one)** units did you drink today? _____ | | | |
| How many bottles of the provided **spring water** did you drink today? _____ | | | |
| How many cans of the provided **sparkling water** did you drink today? _____ | | | |
| **Please check all of the following that apply to today:** | | | |
| **❑ I ate or drank ONLY the foods and beverages provided by the Metabolic Kitchen.** | | | |
| **❑ I did NOT add salt, pepper, spices, or other condiments.** | | | |
| **❑ I did NOT take any medications, supplements, or other remedies.** | | | |
| **❑** *I either ate or drank non-study foods/beverages; added salt, pepper, spices, or other condiments; or took medication, supplements, or other remedies (please turn this checklist over and follow the prompts).* | | | |
| **❑** *I experienced adverse events (e.g. headache, gas, diarrhea, nausea, etc.). Please tell us more about these events (additional space is on the back).__________________________________________________* | | | |

**Supplemental Table 1** (contd.)

[Back]

| ***Use the “How do I estimate portion sizes” insert to provide details for any non-study foods/drinks***  *(we have included some examples to help you see the kinds of details that we seek)* | | | | |
| --- | --- | --- | --- | --- |
| **Time** | **Foods/drinks/meds/supplements/other consumed** | **Amount/**  **Serving Size** | **Description (e.g. brand, restaurant)** | **Prepared How**  **(e.g., baked, fried)?** |
| *7:30 am* | *Cereal* | *¾ cup* | *Cheerios, Honey-Nut* | *Bowl* |
|  | *Milk* | *½ cup* | *2% Berkeley Farms* | *Added to cereal bowl* |
|  | *Coffee* | *Tall* | *Peet’s regular, black* | *Mug* |
|  | *Half-and-half* | *2 Tablespoons* | *Peet’s coffee counter* | *Added to coffee mug* |
| *2:15 pm* | *Bagel* | *1 each* | *Lenders, White, Onion, 3.5 inch diameter* | *Toasted* |
|  | *Peanut Butter* | *2 Tablespoons* | *Skippy, Regular, Creamy, Salted* | *Added to bagel* |
|  | *Banana* | *½ each* | *Fresh, 7 inches long* | *Added to bagel* |
| *8:00 pm* | *Hot Dog* | *~ 1 inch bite* | *Nathan’s, Jumbo, Beef, no bun, no condiments* | *Took bite while cooking* |
| *9:00 pm* | *Wine* | *4 fluid ounces* | *White Zinfandel (used liquid measuring cup)* | *Wine glass* |
| *9:00 pm* | *Fish Oil Supplements* | *2 softgels* | *Nature Made, 1000 mg (300 mg omega-3) per 2 softgels* | *Took with wine* |
| *9:00 pm* | *Ibuprofen* | *1 tablet* | *200 mg ibuprofen per tablet* | *Took with wine* |

**Supplemental Table 2**: Spearman’s rho (ρ**)** and corresponding p values that showed significant associations between HEI score (total or sub-components) and glycosylation status of serum proteins at baseline in women (n=44) at risk for cardiometabolic disease.

| **HEI/Glycosylation status** | **Total non-fucosylated** | | **Total non-sialylated** | | **Total poly-fucosylated** | | **Total polysialylated** | |
| --- | --- | --- | --- | --- | --- | --- | --- | --- |
|  | **ρ** | **p** | **ρ** | **p** | **ρ** | **p** | **ρ** | **p** |
| **Total vegetables** | **0.47** | **0.00** | 0.05 | 0.77 | -0.21 | 0.17 | 0.01 | 0.97 |
| **Greens and beans** | **0.35** | **0.02** | 0.08 | 0.60 | 0.06 | 0.71 | -0.02 | 0.90 |
| **Sea food and plant Protein** | 0.05 | 0.73 | -0.12 | 0.43 | **-0.31** | **0.04** | -0.03 | 0.84 |
| **Total dairy** | 0.16 | 0.30 | **-0.32** | **0.03** | -0.03 | 0.86 | -0.03 | 0.86 |
| **Refined grain** | **0.44** | **<0.01** | **-0.34** | **0.02** | **-0.53** | **<0.01** | **0.31** | **0.04** |
| **Total score** | 0.29 | 0.06 | -0.21 | 0.18 | **-0.42** | **0.01** | 0.12 | 0.45 |
